# Supplementary material for: Screening and Structural Characterization of Heat Shock Response Elements (HSEs) in Entamoeba histolytica Promoters
Source: Int J Mol Sci. 2024 Jan 21;25(2):1319. doi: 10.3390/ijms25021319 (PMC10815948; doi:10.3390/ijms25021319)
Supplement: Supplementary file 1 [file ijms-25-01319-s001.zip › Supplementary image captions.pdf]

Supplementary Figure S1. Ontology of E. histolytica genes with HSE elements. HSE Ehpgp5 (blue bar), HSE Ehsp100 (green bar), HSE EhrabB4 (pink bar), HSE EhrabB1 (orange bar), HSE EhrabB5 (red bar) and HSE Ehmlbp (yellow bar).

Supplementary Figure S2. Ontology of genes with HSE elements of E. histolytica in functional groups. a) molecular functions, b) biological processes and c) protein classes.

Supplementary Figure S3. 3D structural modeling and validation of tEhDBD5 and tEhDBD6. (a) Creation of the tEhDBD5 3D model, followed by exhaustive structural validation using various tools, including (b) PDBsum for Ramachandran plot analysis. The letters A, B, and L highlight the Most Favoured Regions. The lowercase a, b, l, and p mark the Additional Allowed Regions, while the prefixed tilde (~a, ~b, ~l, ~p) points to the Generously Allowed Regions. The designation XX is used for the Disallowed Regions. Additionally, a value of -1 is assigned to a torsion angle located in a non-permissible region. (c,d) Prosa-web was used for the energy profile evaluations, while ERRAT (e) was employed for error quantification, with an * denoting the error value. Yellow bars indicate amino acids with structural errors within a margin of error between 95 and 99%, and red bars identify errors exceeding 99%. (f) VERIFY 3D for sequence-structure compatibility assessments. (g) Creation of the tEhDBD5 3D model, followed by exhaustive structural validation using various tools, including (h) PDBsum for Ramachandran plot analysis. The letters A, B, and L highlight the Most Favoured Regions. The lowercase a, b, l, and p mark the Additional Allowed Regions, while the prefixed tilde (~a, ~b, ~l, ~p) points to the Generously Allowed Regions. The designation XX is used for the Disallowed Regions. Additionally, a value of -1 is assigned to a torsion angle located in a non-permissible region. (i,j) Prosa-web was used for the energy profile evaluations, while ERRAT (k) was employed for error quantification, with an * denoting the error value. Yellow bars indicate amino acids with structural errors within a margin of error between 95 and 99%, and red bars identify errors exceeding 99%. (l) VERIFY 3D for sequence-structure compatibility assessments.

Supplementary Figure S4. Intermolecular analysis. (a) tEhDBD5 and (b)tEhDBD6
