# Supplementary figures and images for: Screening and Structural Characterization of Heat Shock Response Elements (HSEs) in Entamoeba histolytica Promoters
Source: Int J Mol Sci. 2024 Jan 21;25(2):1319. doi: 10.3390/ijms25021319 (PMC10815948; doi:10.3390/ijms25021319)

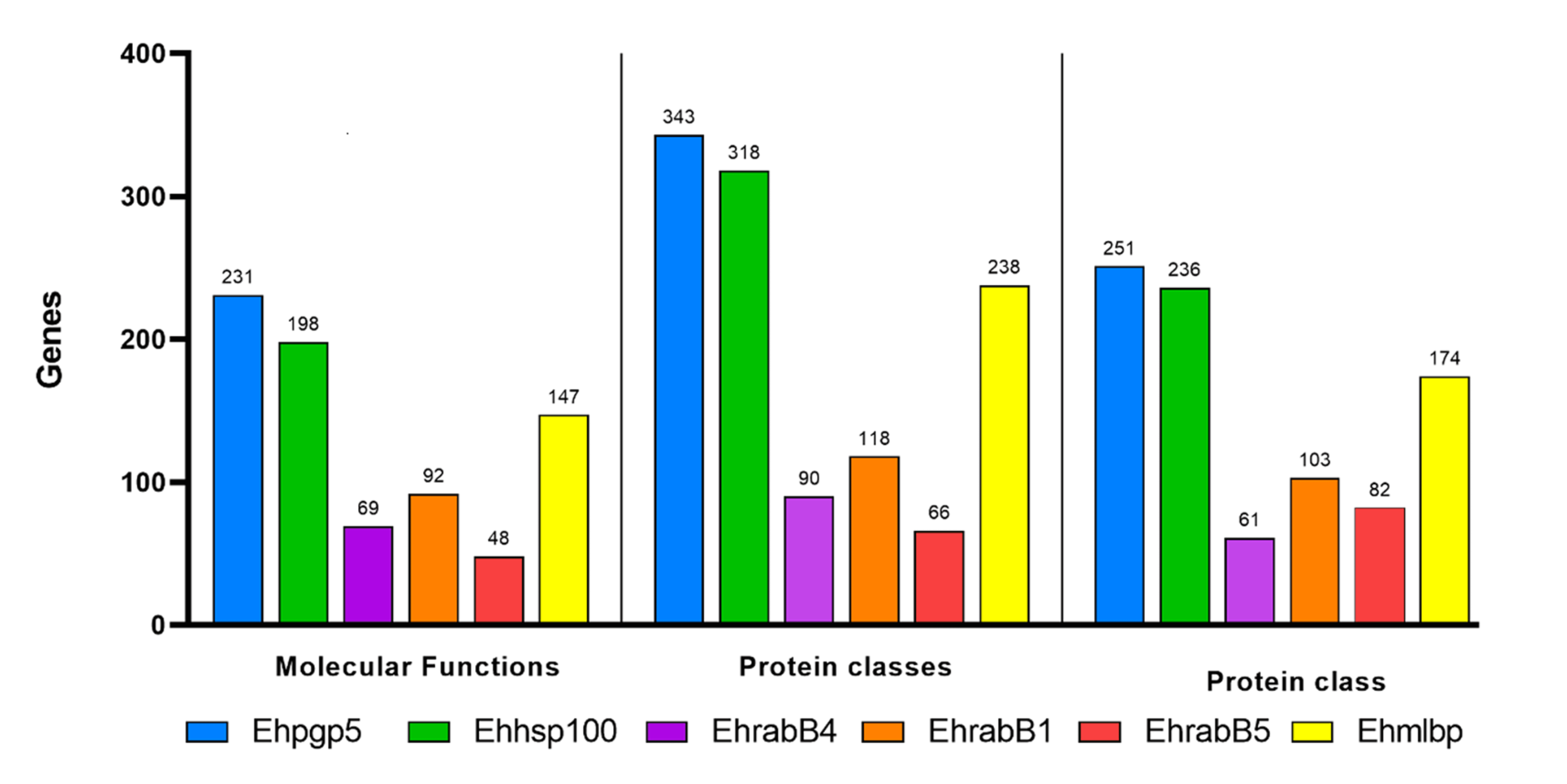

Supplement: Supplementary file 1 [file ijms-25-01319-s001.zip › Supplementary Figure S1.png]
